# The Annual Dinner of the American Dental Society of Europe

**Published:** 1885-11

**Authors:** 


### THE ANNUAL DINNER OF THE AMERICAN DENTAL SOCIETY OF EUROPE.

BY A MEMBER.

The primary object of the American Dental Society of Europe, as of every other dental organization aspiring to the name of a scientific society, is the promotion, advancement, and dissemination of those things which tend to increase the excellence and standing of the profession. At the same time, the privilege of becoming acquainted with members of the profession, who, on account of the great distances which separate them, would otherwise remain strangers, is one which is fully appreciated.

It has long been the custom at the annual meeting of this Society to set aside one afternoon or evening to a purely social meeting, and this part of the programme, with the accompanying dinner, has become as permanent as the discussions themselves, and is regarded as absolutely essential to the success of the meeting.

We leave it for others to judge as to the success of the late meeting from a scientific point of view; the social and gastronomical part of the programme were, we think, all that could be desired. Of course the regular toasts came on in due time. Dr. Cohen welcomed the guests. Dr. Miller eulogized the Kaiser and America, the "Home of Dentistry," and Dr. Sachs remembered the absent members; he "saw a great many who were not there."

But the American Dental Society of Europe counts among its members a "funny man," who never fails to keep the ball rolling and everybody laughing. When Dr. Patton gets up, those who know him prepare to laugh. Dr. Patton toasted the A. D. S. E. somewhat as follows :

*Mr. President and Gentlemen:*

I am pleased to be *able* to rise at this late hour of the festivities, to propose a toast which cannot and should not be forgotten. I might say, as is usual under similar circumstances, "unaccustomed as I am to public speaking" etc., but a morbid originality prevents me. I say I am pleased to be *able* to rise on this occasion; whether I may be able to rise *to* it I know not. Gentlemen, the deeds and achievements of the Anglo-Saxon race, so memorable in history, exert, and will ever exert, that influence on their descendants which causes them to be the leading spirits in all great enterprises. This spirit has led them to be the germs of civilization wherever they could get a foothold, and each continent, yes, the old and new worlds are to-day the proofs of Anglo-Saxon superiority. Our own country, particularly, has to thank these enterprising spirits for its own noble developments. It is an alcoholic success, but, gentlemen, it is a mistake to think that the plucky achievements of our forefathers are not imitated in our own so-called prosaic times; they exist in even more developed exertion, and are merely lost sight of in the overwhelming preponderance of innumerable great enterprises, emanating from scientific advancement.

The bold crusaders of old, armed merely with the weapons of their knightly panoply and calling, rode bravely forth on their long pilgrimages to the East, imbued with the love of adventure, and, upheld by the strong tenets of their faith, fought their way through Saracenic hordes to the shrine of their desires, to return later (when so fortunate) with a retinue, and laden with the spoils of victory gained by their indomitable courage and strong arms.

But, as I said, in these prosaic times we have also our heroes, who fearlessly journey to the East, single-handed, to seek glory and reap rewards in their calling. Yea, have we not the Knights of the Excavator, armed with the weapons of their calling and engines of destruction—many easily carried in their trouser pockets—who gallantly ship themselves on Cunard, or even other lines, leave the tilling of the ground of America, and, landing on the shores of this continent, singly attack the bacterian hordes of Miller, force them from their most impregnable strong-holds, or enclose them away in exile forever and a day? I tell you, gentlemen, when one of this sort gets a few micrococci by the back hair, the shock alone settles them, and after years of stout warfare against the *Germ*-anic Funge Orali, return (also when fortunate) laden with the spoils of victory, carried as their weapons intrinsically in their trousers or other hidden receptacles.

Here, gentlemen, sits to-night a remnant of these bold bactereers and destroying angels, who destroy but to preserve. Gentlemen, I thank you for your presence, and congratulate you on the discriminating election of officers for the campaign of the ensuing year; you could not have done better, for there were no others left, but I can assure you that under the able guidance of your newly-chosen

President, Dr. Cohen, who, in his turn, will be guided by the Vice-President (who will attend particularly to the vicious propensities of the members), no pains will be spared to insure the success of the next campaign.

As you know, we do not think much of ourselves, and I know you all feel as we do, so that buoyant hope will be emblazoned on our banner, and trusting that a strong muster of the body-guard will support us next year in Frankfurt, I call on you, gentlemen, to raise your glasses and join with me in an enthusiastic and energetic Hip! Hip! Hurrah! to the prosperity of the American Dental Society of Europe.

